# Acknowledgment to Reviewers of *Methods and Protocols* in 2021

**DOI:** 10.3390/mps5010012

**Published:** 2022-01-24

**Authors:** Methods and Protocols Editorial Office

**Affiliations:** MDPI AG, St. Alban-Anlage 66, 4052 Basel, Switzerland

Rigorous peer-reviews are the basis of high-quality academic publishing. Thanks to the great efforts of our reviewers, *Methods and Protocols* was able to maintain its standards for the high quality of its published papers. Thanks to the contribution of our reviewers, in 2021, the median time to first decision was 25 days and the median time to publication was 42 days. The editors would like to extend their gratitude and recognition to the following reviewers for their precious time and dedication, regardless of whether the papers they reviewed were finally published:
Adel Martinez-MartinezJoanna Sztuba-SolinskaAhmed HieawyJoão PinheiroAlberto Lo GulloJoaquin De NavascuesAleksandra KołotaJoe MillwardAlessandro A. SartoriJohn A. Kalef-EzraAlexandre BarbosaJong Kook ParkAlexey BogdanovJosé A. Jiménez HeffernanAlisson Aliel Vigano PugliesJu Hyun ParkAmani GilletteJuergen ReichardtAmulya YaparlaJun Ying LiAnahid Ahmadi BirjandiJunchul David YoonAnastas GospodinovJustyna GodosAndrás BikovKarin G. F. GerritsenAndrej BesseKarsten GülowAndrew SomogyiKarsten SchrobbackAneta WieczorekKatarzyna KuterAnette Gjörloff WingrenKatarzyna OtulakAnita TurnerKátia Cândido CarvalhoAnoop SheshadriKazunari YoshidaAnthony J. BaucumKazushige OshitaAravindhan GanesanKelvin Ian AfrashtehfarAreti StavropoulouKhaled Elsayed AhmedArmando VillaltaKonstantin ChekanovArmin EnsserKumar SauravArunava GhoshLachlan H. ThompsonAtsuo YoshidoLaleh YerushalmiBadreddine DouziLaurence J. WalshBarbara RuaroLaurent CorcosBéla KovácsLeonard L. DobensBruno ChrcanovicLidia SabaterBruno RizzutiLiron KlipcanCalum McHaleLorena PochiniCamelia QuekLucía MonacoCamilla PierellaMagdalena BaymakovaCarina BernardoMałgorzata DudaCarmen MirabelliManuel Sanchez-MartinCaterina Maria GambinoMaria Angela PellegrinoChih-Wei PaiMaria M. UlaszewskaChristopher HellenMaria Pina SerraCorinna GeislerMaria SpletterCorinna PreusseMarianna D′ancaCosimo NardiMarianna KapetanouCristian Iulius SurcelMarianna TzanoudakiDafne Campigli Di GiammartinoMario ZuritaDaniela GradiaMartin GiurfaDaria DziewulskaMary Lou CutlerDebadrita PariaMassimo LibraDenis BourgeoisMauro CastroDenise CorridoreMichelle M. W. FeijenDhiman PalMikhail A. LiskovykhDiego San MauroMing-Lun HsuDinesh JillellaMirza PojskicEduardo OliverMoses AyoolaEdyta PasickaMurielle ÅlundEkaitz Errasti-MurugarrenNatalia CheshenkoElizabeth JohnsonNicola RamacciatiElmer MauritsNing ZhaoEmer FerroNishikant WaseEmma SiesmaaPablo Luna-NevarezEric VachonPallabi K. PalErwin De BruinPaolo CalligariEwa SadowyPaolo CapparèEwa TomaszewskaPatricia Kitsao-WekuloEwelina ZielińskaPeter EsserFaisal JamilPing-Chieh PaoFederica FogacciPiotr WychowańskiGerald ChiPradeep DagurGerard Lee L. SeeQi YuGerardo PellegrinoR. Brent SealeGiorgio LombardoRachael CrewGoran MuricRadoslaw BednarekGrzegorz PrzybylskiRaif YuecelGuoqing WangReema SinghGutemberg AlvesRegina EbertGuy K. GermanRenato GondarHamada ElwanRita HõrakHan Su KimRosângela Schwarz RodriguesHeike RudolphRukshan MehtaHenna MuzaffarSalvatore BocchieriHenri VahabiSébastien LamyHervé ReychlerShannon GrayHongfu YuanShumit SahaIan James MartinsSteven VollmerIeva Meidute-KavaliauskieneSusan MirlohiIvica PelivanSvetla BaykouchevaJacek Bogusław SzmańdaTakahito NakajimaJacinto JardimTatiana S. FrolovaJadwiga HamułkaToshiyuki TakahashiJakob MorgensternTsutomu NakazawaJan KosterWenyang DongJanja TrcekYong GeJiaoju GeYun Han

